# Comparative Outcomes of Amblyopia Treatment in High Astigmatism: Stability and Sustained Improvements

**DOI:** 10.3390/jcm14103577

**Published:** 2025-05-20

**Authors:** Chia-Chen Hsu, Lung-Chi Lee, Hsu-Chieh Chang, Chun-Hao Huang, Ke-Hung Chien

**Affiliations:** 1 Department of Ophthalmology, Tri-Service General Hospital, National Defense Medical Center, Taipei 114, Taiwan; water097978kaohsiung9@gmail.com (C.-C.H.); kidday0205@gmail.com (L.-C.L.); 2 Department of Nursing, Tri-Service General Hospital, National Defense Medical Center, Taipei 114, Taiwan; n3197001@gmail.com; 3 Graduate Institute of Nursing, College of Nursing, Taipei Medical University, Taipei 110, Taiwan; 4 Department of Nursing, Tri-Service General Hospital Beitou Branch, Taipei 112, Taiwan; 5 Department of Ophthalmology, Taichung Armed Forces General Hospital, Taichung 411, Taiwan

**Keywords:** amblyopia, astigmatism, spectacles, child, refractive errors

## Abstract

**Background/Objectives**: Astigmatism is a major risk factor for amblyopia. While optical correction improves visual acuity (VA), the long-term treatment outcomes, particularly in children with high astigmatism, remain understudied. This study aimed to determine the treatment effects, time course, and visual outcomes in children aged 3–7 years with high astigmatism and compared their VA improvement with those with low astigmatism. **Methods**: This retrospective cohort study included 63 children with untreated high with-the-rule astigmatism (≥+2.50 diopters [D]) and 46 with low astigmatism (≤1.50 D). The children with high astigmatism were classified based on their baseline best-corrected VA (BCVA) into good-VA (20/25 or better, *n* = 24), mild-VA-impairment (20/25–20/40, *n* = 19), and amblyopia (20/40 or worse, *n* = 20) groups. The primary outcomes include maximal improvement in mean BCVA post treatment, the cumulative probability of achieving a VA of 20/25 or better, and being within one line of the fellow eye. **Results**: The amblyopia, mild-VA-impairment, good-VA, and low astigmatism control groups showed mean improvements of logarithm of the minimum angle of resolution of 0.36 ± 0.08, 0.15 ± 0.05, 0.03 ± 0.04, and 0.01 ± 0.04, respectively. Post treatment with optical correction and/or patching over a mean duration of 46.61 ± 35.22 weeks, amblyopia resolved in all affected children. In the mild-VA-impairment group, only one child did not respond successfully to the treatment. The mean final VA showed no significant intergroup differences (*p* = 0.115). No amblyopia recurrence was observed at a mean follow-up of 118.33 weeks post resolution. **Conclusions**: Timely optical correction and patching effectively improve VA in children with high astigmatism, achieving outcomes comparable to those with low astigmatism.

## 1. Introduction

The prevalence of astigmatism varies depending on age and ethnicity. The estimated prevalence of astigmatism differs significantly across regions, with rates ranging from 9.8% in Southeast Asia to 27.2% in the Americas [1]. Astigmatism causes image distortion along the affected meridian, which can disrupt normal visual development and lead to amblyopia or other visual deficits if not corrected promptly [2,3,4].

Harvey et al. [3] reported that in children from kindergarten to sixth grade, with ≥1.00 diopter (D) of astigmatism, the mean best-corrected letter acuity decreased by approximately 0.2 logarithm of the minimal angle of resolution (logMAR) compared with that of the age-matched children without astigmatism. Furthermore, the magnitude of astigmatism is linked to visual acuity (VA), with each additional D of astigmatism corresponding to an approximate decrease of 0.1 logMAR.

Optical correction can improve VA in children with astigmatism [5,6]. However, the treatment interval and effectiveness of interventions for astigmatism-related amblyopia and meridional amblyopia remain controversial. Two large-scale prospective studies on the treatment of astigmatism in children have been conducted by Harvey and associates [6,7], focusing on Native American populations with a high prevalence rate of astigmatism. Their findings revealed that eyeglass correction over an average of 4 months did not significantly improve VA in children with astigmatism, aged 3–5 years [7]. Another study including children aged 4–13 years reported a significant improvement in VA following 6 weeks of optical correction; however, their VA remained significantly worse compared with that of children without astigmatism [6]. Dobson et al. [8] reported that preschool spectacle correction improves best-corrected letter recognition acuity but not meridional amblyopia in children with astigmatism by kindergarten.

In children, with-the-rule low-grade astigmatism (≤1.50 D) accounts for over 90% of cases [9]. However, high astigmatism (≥2.50 D), though less common, is independently linked to a higher risk of both unilateral and bilateral amblyopia [10]. Pai et al. [11] further demonstrated that young children with ≥1.50 D of aniso-astigmatism or ≥2.50 D of astigmatism are at increased risk, as both are major amblyogenic factors.

Despite its clinical importance, research investigating visual outcomes in pediatric patients with high astigmatism exhibits notable limitations, particularly concerning long-term outcomes beyond the 1-year mark. This paucity of long-term data are likely attributable to the relatively low prevalence of high astigmatism within the general population [2,12]. This study aimed to compare the extent and time course of VA improvement in children with high astigmatism, aged 3–7 years, categorized by baseline VA, with those in children with low astigmatism following treatment with optical correction and patching.

## 2. Materials and Methods

This retrospective study of data focused on children with astigmatism, aged 3–7 years, who visited the Department of Ophthalmology of the Tri-Service General Hospital, Taipei, Taiwan, between June 2012 and January 2022. The protocol and related documents were reviewed and approved by the institutional review board of the Tri-Service General Hospital, Taipei, Taiwan (protocol code A202105131). The requirement for informed consent was waived by the review board owing to the retrospective study design; all data were de-identified. This study was conducted under the Good Clinical Practice guidelines of the Tri-Service General Hospital and according to the Declaration of Helsinki, 1964.

All patients enrolled in this study had visited our Ophthalmology Department for regular follow-ups and underwent complete ophthalmic examinations. All children were treated and followed by a single ophthalmologist (K.-H. Chien) specializing in pediatric ophthalmology and strabismus. Astigmatism was documented in minus-cylinder notation for all patients. Children in the high astigmatism group had with-the-rule astigmatism of ≥2.50 D in either eye, whereas the low astigmatism group comprised children with low or no astigmatism (≤1.50 D in both eyes).

For this study on high astigmatism, children were excluded based on the following criteria: anisohyperopia ≥1.50 D, anisomyopia ≥3.00 D, hyperopia ≥+3.00 D, myopia ≥−3.00 D, or the presence of strabismus, cataract, or other ocular conditions that could impede visual development or reduce best-corrected VA (BCVA) at diagnosis. Children with a previous history of amblyopia treatment, such as refractive correction, patching, or atropine penalization, as well as those aged > 7 years at the initial visit or lost to follow-up, were also excluded.

Additionally, due to the limited number of cases with oblique astigmatism (*n* = 3) and no cases with against-the-rule astigmatism, these subtypes were excluded from the analysis, as their inclusion would have precluded significant statistical evaluation.

**Figure 1:**
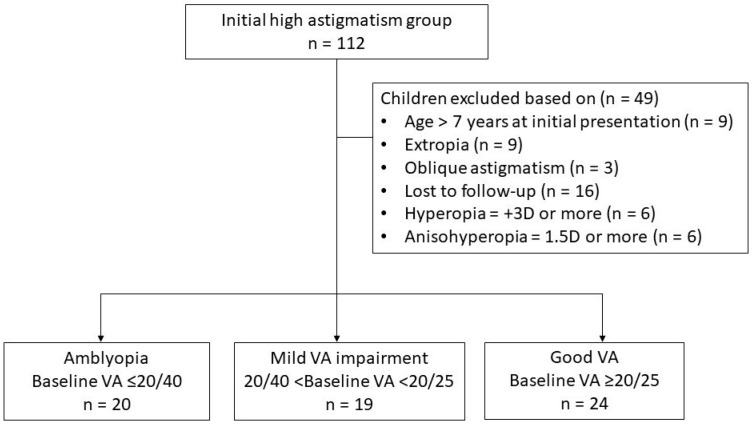
Flow chart of the inclusion process of children with high astigmatism. In total, 112 children were initially assessed, and 49 were excluded based on specific criteria. The remaining 63 children were categorized into 3 groups based on baseline visual acuity (VA): amblyopia (*n* = 20, baseline VA ≤ 20/40), mild-VA-impairment (*n* = 19, 20/40 < baseline VA < 20/25), and good-VA (*n* = 24, baseline VA ≥ 20/25) groups.

A flow chart of patient selection process is illustrated in Figure 1.

In our clinical practice, participants with high astigmatism receive spectacle prescriptions determined by cycloplegic refraction performed with cyclopentolate 1%, with confirmation through retinoscopy and further subjective refinement. Cycloplegia was used to suppress accommodation and ensure accurate assessment of refractive error. Full correction is provided for astigmatism and myopia, whereas hyperopia is corrected in full or symmetrically undercorrected by up to 1.50 D in both eyes.

Correlation between right and left eyes for refractive error and BCVA was evaluated. The intraclass correlation coefficients were 0.841 for spherical power, 0.846 for cylindrical power, 0.917 for baseline BCVA, and 0.912 for final BCVA (all *p* < 0.01), indicating strong interocular agreement [13]. Based on these results, the eye with higher cylindrical power or poorer baseline VA, if both eyes had identical cylindrical power, was selected for the analysis to minimize inter-eye dependency.

Children with high astigmatism were categorized into three subgroups based on their baseline BCVA, in accordance with the Pediatric Eye Disease Investigator Group (PEDIG) criteria [14,15]. Amblyopia was defined as a BCVA of 20/40 (0.3 logMAR) or worse, and successful resolution was defined as achieving a BCVA of 20/25 (0.1 logMAR) or better. Children with a baseline BCVA between 20/40 and 20/25 were classified as having mild VA impairment to better capture intermediate levels of visual dysfunction that did not meet the threshold for amblyopia. Children with a baseline BCVA of 20/25 or better were categorized into the good-VA group. For children with an initial BCVA worse than 20/25, treatment response to spectacles was closely monitored. If no improvement of BCVA was observed over two consecutive follow-ups or within 3 months, patching therapy (1–4 h per day) was initiated. For mild VA impairment, 1 h of patching was prescribed, whereas 2 h were recommended for VA ranging from 20/40 to 20/100 [16,17]. The patching dosage was adjusted if amblyopia proved resistant, guided by the doctor’s discretion for each case, in accordance with the guidelines of the PEDIG [16,18]. Additionally, during the follow-up period, modifications to spectacle correction were made if no sustained improvement in VA was observed over three consecutive follow-ups.

Successful treatment with refractive correction and patching was defined as achieving a VA of 20/25 or better (≥0.10 logMAR) and being within one line of the fellow eye. Children with initial BCVA worse than 20/25 were assessed every 1–3 months to monitor VA improvement. Follow-up assessments were conducted 6–12 months following successful treatment. If a child showed no VA improvement over six consecutive follow-ups, the condition was considered stabilized.

Children with high astigmatism and good VA as well as those with low astigmatism were monitored at least every 6 months. This was facilitated by the government-supported Taiwan Student Vision Care Program, which provided funding to establish centers dedicated to children’s vision care.

Data regarding VA with or without spectacles and cycloplegic refractive errors, including spherical and cylindrical errors (dioptric power and axis), were collected for analysis. Cycloplegic refractive errors in children were measured using a TONOREF III auto-refractor (Nidek Co., Ltd., Aichi, Japan) 30 min after administering 1% cyclopentolate eye drops (Cyclogyl; Alcon Labs, Fort Worth, TX, USA).

VA was evaluated using the ETDRS Tumbling E Chart, and the obtained results were converted to logMAR values for statistical analysis. Normality of the data were evaluated using the Shapiro–Wilk test due to the small sample sizes within each subgroup (Table S1). As most variables were found to deviate from a normal distribution, nonparametric statistical methods were subsequently employed. Differences in the distribution of VA, cylindrical power, time to achieve successful treatment, and final VA among three or more groups were analyzed using the Kruskal–Wallis test. If the test result was significant, the Mann–Whitney U test was subsequently performed to identify which sample pairs exhibited significant differences. Wilcoxon’s signed rank test was applied to assess whether the treatment had a significant impact by comparing the mean change in VA over time in the amblyopia and mild-VA-impairment group. Statistical analysis was performed using SPSS software (version 26.0 for Windows; SPSS Inc., Chicago, IL, USA). All continuous variable data within each group were presented as the mean ± standard deviation (SD). In this study, a *p*-value < 0.05 was considered statistically significant.

Given the limited sample size, the effect size was estimated using G*Power software (Version 3.1.9.7; Heinrich-Heine-Universität Düsseldorf, Germany). Effect sizes were interpreted according to Cohen’s standards, with values of f ≥ 0.40 considered indicative of a large effect [19].

## 3. Results

### 3.1. Baseline Characteristics

Sixty-three children with high with-the-rule astigmatism (≥2.50 D in either eye) were included in the study. Among them, 24 children had good initial VAs (20/25 or better), 19 had mild VA impairment (20/40 to 20/25), and 20 were diagnosed with amblyopia (20/40 or worse). The low astigmatism group comprised 46 children with low or no astigmatism (≤1.50 D in both eyes). The baseline clinical characteristics of these patients are presented in Table 1 and Table S2. Male participants constituted 50%, 57.9%, 35%, and 50% of the good-VA, mild-VA-impairment, amblyopia, and low astigmatism groups, respectively. The mean (±SD) ages were 5.03 (±1.00), 4.96 (±0.98), 4.51 (±0.88), and 5.89 (±1.15) years in the good-VA, mild-VA-impairment, amblyopia, and low astigmatism groups, respectively. Among children with high astigmatism, no significant difference in mean age (*p =* 0.142) or cylindrical power (*p =* 0.177) was observed. However, children in the low astigmatism group were generally older than those in the high astigmatism group (*p <* 0.01).

### 3.2. Rate of Successful Treatment and Treatment Duration

**Figure 2:**
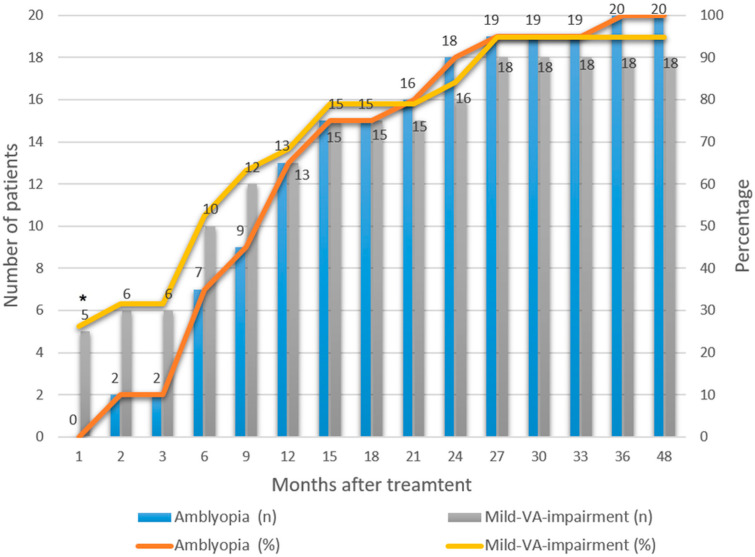
Numbers and percentages of children who achieved successful treatment outcomes over time. Numbers and cumulative percentages of patients in the amblyopia group and the mild-VA-impairment group achieving successful treatment over time. m, months; VA, visual acuity. * *p* value < 0.05, obtained from the chi-square test for difference in cumulative percentages.

Amblyopia resolved in all 20 children, with cumulative resolution rates of 0%, 10%, 35%, 65%, 75%, 90%, and 100% at 1, 3, 6, 9, 12, 24, and 36 months, respectively (Figure 2). The median time to achieve successful treatment was 37.5 weeks, and the final VA was achieved at a median of 57.77 weeks after presentation. Notably, in two children, amblyopia was resolved at 24–36 months, and three achieved their final VA during the same time period.

In the mild-VA-impairment group, the cumulative probability of achieving a VA of 20/25 was 26.3% at 1 month, 31.6% at 3 months, 52.6% at 6 months, 68.4% at 1 year, 84.2% at 2 years, and 94.7% at 2.5 years (Figure 2). One child in this group did not achieve a VA of 20/25. This child presented with a cylindrical power of −5.5 D and an initial VA of 0.22 logMAR, which improved to 0.16 logMAR after 14 months of treatment but showed no further improvement by 23 months, at the age of 6.2 years.

In this study, no recurrence of amblyopia was observed in the 20 children, with a mean age of 8.19 ± 2.39 years, followed for a median of 66.79 weeks after amblyopia resolution. No significant difference was observed between the amblyopia and mild-VA-impairment groups in terms of mean age (*p =* 0.482), treatment duration required to achieve a VA of 20/25 (*p =* 0.249), or the follow-up period after reaching a VA of 20/25 (*p =* 0.978) (Table 2). Over 1 year of follow-up was attained in 70% of the amblyopia group and 63.2% of the mild-VA-impairment group.

For children with high astigmatism and good initial VA, despite the absence of amblyopia, spectacles were prescribed, and the final VA was attained within a median of 3.93 weeks of treatment (Table 2).

### 3.3. Changes in VA over Time

A significant difference in the mean (±SD) baseline BCVA (logMAR) at the initial presentation was observed across all the groups (*p <* 0.001). Even among children with high astigmatism who had good VA, the BCVA was lower than that in those with low astigmatism (Table 2). The average final VA was compared across all groups and showed no significant difference (*p =* 0.115); the average final VA (logMAR) was 0.02 ± 0.04, 0.02 ± 0.05, 0.01 ± 0.04, and 0.01 ± 0.02 in the good-VA, mild-VA-impairment, amblyopia, and low astigmatism groups, respectively. The effect size (Cohen’s f = 0.841) for the change in BCVA, calculated from the group means and pooled standard deviation, indicated a large effect according to Cohen’s classification.

#### 3.3.1. VA Improvement over Time Compared with the Baseline Values

**Figure 3:**
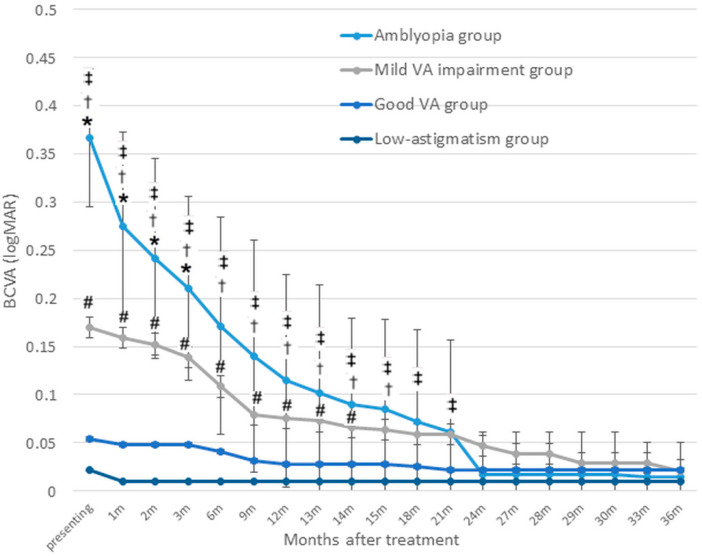
Difference in visual acuity improvement between the groups. Visual acuity (VA, logMAR) improvement over treatment time is shown. BCVA, best-corrected visual acuity; VA, visual acuity; logMAR, logarithm of the minimal angle of resolution; m, months. * significant difference in VA between the amblyopia and mild-VA-impairment groups; † significant difference in VA between the amblyopia and good-VA groups; ‡ significant difference in VA between the amblyopia and low astigmatism groups; # significant difference in VA between the mild-VA-impairment and good-VA groups (*p* < 0.05).

Figure 3 illustrates the changes in VA (logMAR) across the four groups. In the amblyopia group, the mean VA improved significantly from 0.37 ± 0.07 at baseline to 0.27 ± 0.10 at 1 month, 0.21 ± 0.10 at 3 months, 0.17 ± 0.11 at 6 months, 0.11 ± 0.11 at 12 months, 0.02 ± 0.04 at 24 months, and 0.01 ± 0.04 at 36 months (all *p <* 0.01, Wilcoxon’s signed rank test).

Among children with mild VA impairment (baseline mean 0.17 ± 0.03), VA showed a slight but non-significant improvement within the first 3 months, followed by a significant gain at 6 months (0.11 ± 0.07, *p* = 0.003). Further improvements were observed at 12 months (0.075 ± 0.07), 24 months (0.05 ± 0.06), and 36 months (0.02 ± 0.05) (all *p* < 0.01).

In the high astigmatism group with good baseline VA (mean 0.05 ± 0.05), VA remained stable during the early treatment period and showed a significant improvement after 9 months (0.03 ± 0.04, *p* = 0.02), with further gains reaching a final VA of 0.02 ± 0.04 at 21 months (*p <* 0.01).

#### 3.3.2. Intergroup Comparisons

The VA in the amblyopia group did not significantly differ from that of the mild-VA-impairment group at 6 months post treatment (*p* = 0.13), the good-VA group at 18 months post treatment (*p* = 0.08), or the low astigmatism group at 24 months post treatment (*p* = 0.51). Similarly, the VA in the mild-VA-impairment group was comparable to that of the initial good-VA group at 15 months post treatment (*p* = 0.11) and the low astigmatism group at 36 months post treatment (*p* = 0.21; Figure 3).

The mean VA improvement for children in the amblyopia group was 2.5 lines at 1 year and 3.5 lines at 2 years, which was significantly greater than the improvements in the other three groups (*p <* 0.001).

For children with an initial mild VA impairment, the mean improvement was 0.9 lines at 1 year and 1.2 lines at 2 years, which was significantly greater than the improvements observed in the good-VA and low astigmatism groups (*p* < 0.001). However, the VA improvements were not significantly different between the high astigmatism with good VA group and the low astigmatism group at 1 year and 2 years.

#### 3.3.3. Trends and Plateau of Changes in VA

Figure 3 also demonstrates the trend in BCVA improvement, which differed significantly across the groups post treatment. The amblyopia group demonstrated the steepest improvement in VA during the first month. After the first month, the rate of improvement slowed slightly but continued to increase steadily, leading to substantial gains by 12 months. Beyond 12 months, the rate of improvement began to slow, with the trend line gradually flattening. From 24 months onward, the improvement trend plateaued. In contrast, the initial mild-VA-impairment group exhibited a moderate initial improvement, with a shallower slope, compared with the amblyopia group. Between 3 and 9 months, the rate of improvement seemed to increase slightly, as reflected by a steeper slope during this phase. After 9 months, the rate of improvement began to slow, with the trend line flattening gradually. By around 12 months or later, the improvement appeared to plateau. The good-VA and low astigmatism groups showed minimal changes over time.

### 3.4. Comparison Between the Patching and No-Patching Subgroups

In subgroup analysis, we stratified children in the amblyopia and initial mild-VA-impairment groups into a patching group of sixteen children and a no-patching group of twenty-three children (Table S3). Nine (45%) and seven children (36.8%) in the amblyopia and initial mild-VA-impairment groups received patching, respectively. In the patching group, four children underwent patching for 4 h per day, whereas the remaining children received patching for 1–2 h per day. No patient underwent patching for ≥6 h per day. The mean (±SD) patching duration was 34.37 (±26.72) weeks in the patching group. The baseline and final BCVA were lower in the patching group than in the no-patching group, although these differences were not significant (*p* = 0.512 and 0.058, respectively). No significant difference was found between the two groups in terms of sex, age at diagnosis, age at successful treatment, age of final VA, spherical power, cylindrical power, baseline BCVA, or final VA.

## 4. Discussion

Astigmatism impairs vision development and, without proper treatment, may lead to amblyopia. This retrospective study evaluated the long-term outcomes following treatment in children with high astigmatism. In previously untreated children with amblyopia, VA improved by an average of 3.5 lines following treatment over a mean duration of 16.4 months. Treatment with optical correction and/or patching resulted in complete resolution of amblyopia in all patients, with a median resolution time of 37.5 weeks. No significant difference in final VA was observed among children with high astigmatism and good VA, mild VA impairment, amblyopia, and low astigmatism.

In the amblyopia group, 65% and 90% of children experienced resolution of amblyopia at 12 and 24 months, respectively. These results are consistent with those of a previous study, in which 74% of 113 children with untreated bilateral refractive amblyopia (hypermetropia and astigmatism) achieved binocular VA of 20/25 or better after 52 weeks of treatment [12]. The mean final VA (logMAR) in the amblyopia group was 0.01 at 36 months, which was slightly better than the values 0.11–0.15 reported in previous studies involving children with hypermetropic, astigmatic, anisometropic, and strabismic amblyopia undergoing treatment with optical correction and additional use of patching, atropine, or both [12,20]. This difference may be because better baseline VA can lead to better VA outcomes [12,21]. Although children with ≥2.50 D astigmatism were particularly deemed to have amblyogenic factors in previous studies [11,22], our study revealed that their VA outcomes and amblyopia resolution rates were comparable to those of children with amblyopia due to other etiologies, suggesting that the management of amblyopia caused by high astigmatism could be similarly effective.

Notably, the trend in VA improvement varied significantly among the different groups following treatment. The amblyopia group exhibited the most prominent improvement during the first month, reflecting significant early visual recovery. This aligns with findings from previous studies on children with astigmatic, anisometropic, strabismic, or bilateral refractive amblyopia, which demonstrated that the majority of improvements with optical correction take place within the initial weeks of treatment [12,20,21,23,24]. After the first month, the rate of improvement slowed slightly but continued to increase steadily up to around 12 months, showing noticeable progress, consistent with the findings of Wallace et al. [12], who reported that children with bilateral refractive amblyopia achieved a mean improvement of 2.3 lines within the first 5 weeks, out of a total mean improvement of 3.9 lines at 1 year, demonstrating that visual gains became less prominent after the first month. Beyond 12 months, the rate of improvement slowed further, with the trend line gradually flattening, and VA stabilizing by 24 months.

In contrast, the mild-VA-impairment group demonstrated a more complex pattern, showing moderate initial improvement during the first 3 months, followed by an accelerated improvement between 3 and 9 months, and then a gradual slowdown after 9 months, eventually reaching a plateau earlier than the amblyopia group did. This unique progression suggests that the mild-VA-impairment group benefitted significantly from the consistent use of spectacles during the intermediate phase, highlighting an extended window for potential therapeutic interventions.

The high astigmatism with good VA and low astigmatism groups exhibited minimal changes in VA over the study period. Their trend lines remained relatively flat due to their near-optimal baseline VA. These findings underscore the importance of tailoring interventions based on the characteristics of each group. For children with amblyopia, the early phase is critical for visual recovery, with sustained treatment over 2 years leading to maximal visual gains. Meanwhile, the mild-VA-impairment group may require focused attention during the intermediate phase to optimize the outcomes.

The amblyopia group showed continued VA improvements for up to 2 years, which stabilized thereafter. This finding differs from that reported by Harvey et al. [6], who observed no additional treatment effect beyond 6 weeks to 1 year in children treated solely with optical correction. This discrepancy may be attributed to variations in ethnicity, treatment protocols, and follow-up durations. However, Lin et al. [25] reported that the continual wearing of spectacles in children with highly refractive amblyopia led to gradual VA improvement until 18 months, which was important for achieving maximal VA. Other studies have also reported VA improvement at around 1 year of treatment and beyond with spectacle correction [21,26,27]. Thus, the results of our study support the notion that continuous spectacle correction is crucial for achieving maximal VA in children with astigmatic amblyopia. In this study, although one child did not achieve a VA of 20/25 after 23 months of treatment, others (*n* = 3) achieved their final VA between 24 and 36 months, highlighting the potential for further improvement with extended treatment.

The high rate of treatment success observed in our study may be partly attributed to the inclusion of patching therapy for some patients. Among children with an initial VA worse than 20/25, 41% received occlusion therapy when VA improvement plateaued after two consecutive follow-up visits or within 3 months of refractive correction. The time to treatment success and final VA outcomes were comparable between the patching and no-patching groups. While these exploratory findings suggest a potential association between combining spectacles with patching and improved visual outcomes in high astigmatism-related visual impairment, causality cannot be inferred due to the retrospective design and limited sample size. Further prospective studies with larger cohorts are warranted to confirm these observations. Wallace et al. [12] similarly demonstrated the benefits of combining therapies for bilateral refractive amblyopia, with patching or atropine added in 12% of cases where VA stagnated, achieving an 86% probability of binocular VA of 20/25 or better at 52 weeks. Additionally, a randomized trial involving 507 children showed significantly higher response rates (≥2-line VA improvement) with combined treatment (53%) versus spectacle correction alone (25%) (*p* ≤ 0.001) [28]. These findings underscore the potential value of integrating occlusion therapy with refractive correction to enhance treatment outcomes.

The literature concerning the recurrence rate and contributing factors for astigmatism-related amblyopia is limited. We observed no instances of amblyopia recurrence within the amblyopia group during an average follow-up of 2.3 years. However, the 100% amblyopia resolution rate may represent a potential overestimate and should be interpreted with caution due to the small sample size, retrospective study design, and limited follow-up duration. Previous studies have reported amblyopia recurrence in approximately one-quarter of children with strabismic or anisometropic amblyopia, even when VA reaches normal levels within the first year following treatment cessation [29,30]. In contrast, children with high astigmatism appear to exhibit lower rates of amblyopia recurrence or regression after successful treatment. Continuous spectacle wear may play a protective role. In our study, children were encouraged to wear spectacles consistently until at least 10 years of age, aligning with recommendations for maintaining visual development [31]. Steele et al. [32] similarly reported a 14% recurrence rate in children with anisometropic amblyopia treated exclusively with spectacle correction over an average follow-up of 1.7 years. Although the differences in study populations and treatment approaches must be considered, these findings suggest that continuous optical correction contributes to greater VA stability in children with refractive amblyopia. Nevertheless, our findings should be validated in larger, prospective studies with longer follow-up periods.

Within the patching group, no VA regression was observed following the cessation of patching. This may be attributed to the relatively low patching intensity employed in our study. High-intensity patching (6–8 h per day) has been identified as a potential risk factor for amblyopia recurrence [29]. None of our enrolled patients underwent more than 4 h of daily patching, and we gradually reduced the patching duration to 2 h per day before cessation.

Despite the 10-year study span (2012–2022), all children were treated and followed by the same ophthalmologist at a single institution, ensuring consistency in clinical evaluation and treatment decisions. While minor variations in clinical judgment may have occurred over time, our institution adhered to a consistent clinical approach for managing patients with high astigmatism, including general principles for spectacle prescription, initiation of patching therapy, and follow-up intervals. Nevertheless, this minor heterogeneity across the 10-year period should be considered when interpreting the study results.

Our study has some limitations. First, the retrospective study design limited the ability to conduct standardized testing, implement randomized controls, and establish rigorous follow-up protocols. Additionally, although post hoc effect size analysis indicated adequate power for the primary outcome, the lack of a priori sample size calculation may still limit the statistical robustness, particularly for subgroup comparisons. While baseline characteristics were comparable between the groups, residual confounding could not be entirely excluded. Treatment allocation (e.g., decisions regarding patching therapy) was based on physician discretion rather than randomization, introducing a potential treatment selection bias. Future prospective, randomized studies are warranted to further validate these findings. Additionally, although astigmatism is recognized to affect VA and other visual functions, such as grating acuity, vernier acuity, contrast sensitivity, and stereoacuity [3,33,34], our analysis was restricted to best-corrected recognition acuity due to incomplete medical record documentation. Additional research is needed to determine whether treatment can improve these other visual functions in children with high astigmatism. Second, only children with untreated high with-the-rule astigmatism who met our strict inclusion criteria were recruited, resulting in a small sample size, especially in the subgroup analyses; thus, the data analysis was limited. Moreover, we analyzed only the eye with the higher cylindrical power or poorer BCVA, which may have led to an overestimation of astigmatism severity and treatment improvement, as well as the introduction of selection bias. Children with against-the-rule and oblique astigmatism were excluded due to their extremely low representation in our cohort, consistent with the natural predominance of with-the-rule astigmatism in Asian pediatric populations [35]. Including these subtypes would have precluded notable statistical analysis and potentially introduced bias. Third, all children in this study had mild (0.16 to 0.22 logMAR) or moderate (0.30 to 0.52 logMAR) visual deficit, which, given the absence of severe amblyopia, might have contributed to more effective treatment and led to a high amblyopia resolution rate.

## 5. Conclusions

This retrospective study demonstrates that children with high astigmatism-related amblyopia can achieve visual outcomes comparable to those of children with low astigmatism following treatment with spectacles and/or patching, along with good stability of treatment effects. The trends in visual acuity improvement vary according to baseline VA, and patching therapy, sustained optical correction, and follow-up beyond 2 years should be considered to maximize treatment outcomes. Continued research using prospective and randomized approaches is needed to validate these outcomes.

## Figures and Tables

**Table 1:** Baseline clinical characteristics of the children.

	High Astigmatism Group				Low Astigmatism Group (*n* = 46)
	Amblyopia (VA ≤ 20/40) (*n* = 20)	Mild VA Impairment (20/40 < VA < 20/25) (*n* = 19)	Good VA (VA ≥ 20/25) (*n* = 24)	*p* Value *	
Sex (male/female)	7/13	11/8	12/12	0.350	23/23
Age (years)					
3 to <4	5	3	3		3
4 to <5	8	6	7		7
5 to <6	6	7	10		11
6 to ≤7	1	3	4		25
Mean (±SD; range)	4.51 (±0.88; 3–6.8)	4.96 (±0.98; 3.6–6.8)	5.03 (±1.0; 3–6.8)	0.142	5.89 (±1.15; 3.1–7)
Cylindrical power (D) ^†^					
0 to <−0.50					17
−0.50 to <−1.00					11
−1.00 to ≤−1.50					18
−2.50 to <−3.50	9	11	14		
−3.50 to <−4.50	6	6	9		
−4.50 to <−5.50	3	1	1		
−5.50 to ≤−6.50	2	1	0		
Mean (±SD; range)	−3.84 (±1.13; −6.50 to −2.50)	−3.34 (±0.82; −5.50 to −2.50)	−3.25 (±0.59; −4.50 to −2.50)	0.177	−0.70 (±0.46; −1.50 to 0)

* *p* value obtained using the chi-square test for difference in sex and Kruskal–Wallis test for difference in the distribution of cylindrical power. ^†^ The eye with greater cylindrical power was chosen for analysis. If both eyes had identical cylindrical power, the eye with the worse best-corrected visual acuity was selected. D, diopters; SD, standard deviation; VA, visual acuity.

**Table 2:** Visual acuity outcomes in the high astigmatism group compared with the low astigmatism group after treatment.

	High Astigmatism Group				Low Astigmatism Group (*n* = 46)
	Amblyopia (VA ≤ 20/40) (*n* = 20)	Mild VA Impairment (20/40 < VA < 20/25) (*n* = 19)	Good VA (VA ≥ 20/25) (*n* = 24)	*p* Value *	
BCVA^†^ at baseline (logMAR), mean (±SD; range)	0.37 (±0.07; 0.30–0.52)	0.17 (±0.03; 0.16–0.22)	0.05 (±0.05; 0.00–0.10)	0.00	0.02 (±0.04; 0.00–0.10)
Final VA (logMAR), mean (±SD; range)	0.01 (±0.04; 0.00–0.10)	0.02 (±0.05; 0.00–0.16)	0.02 (±0.04; 0.00–0.10)	0.32	0.01 (±0.02; 0.00–0.10)
Time to achieve successful treatment (weeks) ^‡^, median (IQR)	37.5 (17.04–72.39)	22.5 (4.00–58.14)		0.25	
Age at treatment success (years) ^‡^, mean (±SD; range)	5.73 (±0.85; 4.6–7.6)	5.75 (±1.4; 4.1–8.8)		0.48	
Time to reach final VA (weeks), median (IQR)	57.77 (34.86–91.39)	38.86 (24–84.71)	3.93 (0–27.5)	0.00	
Time of last follow-up from treatment success (weeks), median (IQR) ^‡^	66.79 (46.36–158.5)	84.29 (24–194.86)		0.98	

* *p* value obtained using Mann–Whitney U test for difference in the distribution of VA. ^†^ The eye with greater cylindrical power was chosen for analysis. If both eyes had identical cylindrical power, the eye with the worse BCVA was selected. ^‡^ The data excluded one patient from the high astigmatism group who failed to achieve successful treatment. BCVA, best-corrected visual acuity; VA, visual acuity; logMAR, logarithm of the minimal angle of resolution; SD, standard deviation; IQR, interquartile range.

## Data Availability

The datasets used and/or analyzed during the current study are available from the corresponding author on reasonable request.

## Supplementary Materials

The following supporting information can be downloaded at https://www.mdpi.com/article/10.3390/jcm14103577/s1: Table S1: Shapiro–Wilk test *p*-values for assessment of normality across groups; Table S2: Spherical power of the groups; Table S3: Subgroup analysis outcomes of the patching and no-patching groups.

## Abbreviations

The following abbreviations are used in this manuscript:

BCVA	Best-corrected visual acuity
logMAR	Logarithms of the minimal angle of resolution
SD	Standard deviation
VA	Visual acuity
