# Supplementary material for: Comparative Outcomes of Amblyopia Treatment in High Astigmatism: Stability and Sustained Improvements
Source: J Clin Med. 2025 May 20;14(10):3577. doi: 10.3390/jcm14103577 (PMC12112649; doi:10.3390/jcm14103577)
Supplement: Supplementary file 1 [file jcm-14-03577-s001.zip › jcm-3508274-supplementary.pdf]

Supplementary Materials

Table S1. Shapiro–Wilk test p-values for assessment of normality across groups

Shapiro–Wilk *P* value†	Amblyopia (VA ≤20/40) (n=20)	Mild VA impairment (20/40 <VA <20/25) (n=19)	Good VA (VA ≥20/25) (n=24)	Low astigmatism (n=46)
Age	0.189	0.322	0.845	0.000
Cylindrical power (D)	0.022	0.004	0.013	0.001
BCVA at baseline (logMAR)	0.000	0.000	0.000	0.000
Final VA (logMAR)	0.000	0.000	0.000	0.000

Abbreviations: BCVA, best-corrected visual acuity; D, diopters; VA, visual acuity; logMAR, logarithm of the minimal angle of resolution

†Shapiro–Wilk test was used to assess the normality of variable distributions in each group.

Table S2. Spherical power of the groups

Spherical power (D)	Amblyopia (VA ≤20/40) (n=20)	Mild VA impairment (20/40 <VA <20/25) (n=19)	Good VA (VA ≥20/25) (n=24)	Low astigmatism (n=46)
-2.50 to <-2.00				2
-2.00 to <-1.50				
-1.50 to <-1.00				7
-1.00 to <-0.50				4
-0.50 to <0.00				8
0.00 to <+0.50	13	5	13	15
+0.50 to <+1.00	0	5	3	3
+1.00 to <+1.50	4	1	4	5
+1.50 to <+2.00	1	4	1	2
+2.00 to <+2.50	1	1		
+2.50 to ≤+3.00	1	3	3	

Abbreviations: D, diopters; VA, visual acuity

Table S3. Subgroup analysis outcomes of the patching and no-patching groups

Characteristic	Patching group (n=16)	No-patching group (n=23)	*P* value[†]
Sex (male/female)	5/11	13/10	0.192
Age at diagnosis (years), mean (±SD; range)	4.63 (±0.93; 3–6.8)	4.8 (±0.97; 3.2–6.8)	0.646
Age at treatment success (years)[§], mean (±SD; range)	5.58 (±0.97; 3.8–7.6)	5.66 (±1.25; 4.2–8.75)	0.668
Age at achieving final VA (years), mean (±SD; range)	5.73 (±0.83; 4.1–6.9)	6.26 (±1.12; 4.08–8.9)	0.259
Time to treatment success (weeks)[§], mean (±SD; range)	50.82 (±35.76; 4–102)	37.86 (±35.59; 2–102)	0.204
Cylindrical power (D)[‡], mean (±SD; range)	−3.73 (±1.2; −6.5 to −2.50)	−3.50 (±0.86; −5.0 to −2.5)	0.618
Spherical power (D)[‡], mean (±SD; range)	0.73 (±0.91; 0–3.0)	0.99 (±1.28; 0–3.0)	0.614
BCVA[‡] at baseline (logMAR), mean (±SD; range)	0.28 (±0.11; 0.16–0.52)	0.26 (±0.12; 0.16–0.52)	0.512
Final VA (logMAR), mean (±SD; range)	0.03 (±0.05; 0.00–0.16)	0.01 (±0.02; 0.00–0.10)	0.058
Cylindrical power (D)			
−2.50 to <−3.50	8	12	
−3.50 to <−4.50	5	7	
−4.50 to <−5.50		4	
−5.50 to ≤−6.50	3		
Spherical power (D)			
0.00 to <+0.50	8	10	
+0.50 to <+1.00	3	2	
+1.00 to <+1.50	1	4	
+1.50 to <+2.00	2	4	
+2.00 to <+2.50	1		
+2.50 to ≤+3.00	1	3	

Abbreviations: D, diopter; BCVA, best-corrected visual acuity; VA, visual acuity; logMAR, logarithm of the minimal angle of resolution; SD, standard deviation

[†]*P* value obtained using the chi-square test for difference in sex and Mann–Whitney U test for difference in the distribution of age, VA, cylindrical power, and time to reach amblyopia resolution and final VA.

[‡]The eye with greater cylindrical power was chosen for analysis. If both eyes had identical cylindrical power, the eye with the worse BCVA was selected.
